# Somatic Changes of Maternal High-Fat Diet on Offspring—Possible Deleterious Effects of Flavonoids?

**DOI:** 10.3390/nu16234022

**Published:** 2024-11-24

**Authors:** Cristina Mihaela Ormindean, Razvan Ciortea, Carmen Elena Bucuri, Andrei Mihai Măluțan, Cristian Ioan Iuhas, Ciprian Gheorghe Porumb, Renata Lacramioara Nicula, Vlad Ormindean, Maria Patricia Roman, Ionel Daniel Nati, Viorela Suciu, Adrian Florea, Carolina Solomon, Madalina Moldovan, Dan Mihu

**Affiliations:** 1 2nd Department of Obstetrics and Gynecology, “Iuliu Haţieganu” University of Medicine and Pharmacy, 400012 Cluj-Napoca, Romania; cristina.mihaela.prodan@gmail.com (C.M.O.); cbucurie@yahoo.com (C.E.B.);; 2 Department of Cell and Molecular Biology, Faculty of Medicine, “Iuliu Haţieganu” University of Medicine and Pharmacy, 400349 Cluj-Napoca, Romania; 3 Radiology Department, “Iuliu Hațieganu” University of Medicine and Pharmacy, 400347 Cluj-Napoca, Romania; 4 Department of Physiology, “Iuliu Haţieganu” University of Medicine and Pharmacy, Clinicilor Street, No. 1, 400006 Cluj-Napoca, Romania

**Keywords:** obesity, pregnancy, adipokines, animal model, flavonoids, antioxidants, ultrastructural modifications

## Abstract

**Background/Objectives**: The rapidly increasing rate of obesity has become an extremely important public health problem, particularly in developed countries. Obesity is associated with a range of health problems, often referred to as the metabolic syndrome. Adipose tissue is now regarded as an endocrine organ responsible for the hormonal secretion of adipokines, which are cytokines involved in various physiological processes. It has been established that adipokines play a key role in the regulation of many processes in the human body. The aim of the current study was to use an animal model to investigate the possible influence of obesity and adipokines on the gestational period, on the development of offspring, and to assess whether these changes are influenced by the administration of antioxidant agents and flavonoids. **Methods**: The present study was performed using 5 groups of 7 female Wistar albino rats. A control group was used to which a 5% lipid diet was administered, and the other 4 groups were fed an obesogenic 65% lipid diet. From the 4 groups that received obesogenic diet one group received no supplement, and the rest of 3 received Detralex, Sel-E-Vit and Rutin (antioxidants and flavonoids). Study times for both pregnant groups and offsprings: on day 15 of gestation, venous blood was drawn to determine adipokine (leptin and visfatin) levels; on days 18–22 ultrasound examination was performed to measure the thickness of adipose tissue in the abdominal wall; for each batch a number of 10 offspring were selected for the measurements (pup weight, brain weight, head length, head width, spine length, width between shoulder blades, coxal bone length), adipokine levels in the offspring (from brain tissue) were also determined, as well as the existence of changes in the brain tissue of the offspring identified by electron microscopy. **Results**: The results of the study showed that the high-fat diet (HFD) led to a significant increase in body weight and abdominal wall thickness in pregnant females compared to the control group. The levels of leptin and visfatin were also affected by the HFD, with leptin levels being significantly higher in the HFD group and visfatin levels being lower. In the offspring, the HFD group had a significantly higher body mass and brain weight compared to the control group. The anthropometric measurements of the offspring were also affected by the maternal diet, with the HFD group having larger dimensions overall. Interestingly, the offspring of the groups that received flavonoids in addition to the HFD had significantly smaller dimensions compared to both the HFD group and the control group. **Conclusions**: The results of this experimental study reinforce what is already known about the effects of obesity on the gestation period and offspring and at the same time, the current study highlights the existence of possible adverse effects of flavonoid compounds on the development of pregnancy and offspring, opening the way for future studies on the benefits and risks of using these compounds during gestational period.

## 1. Introduction

The rapidly increasing rate of obesity has become an extremely important public health problem, particularly in developed countries [1]. Obesity has been ranked by the World Health Organization as among the top 10 health risk conditions and in top 5 among developed countries [2]. According to the most recent data, 30.5% of women in Europe are overweight (body mass index (BMI) > 25 kg/m^2^) and a staggering 15.9% of them are classified as obese (BMI > 30 kg/m^2^) [3,4]. Obesity is associated with a range of health problems, often referred to as the metabolic syndrome, involving the development of insulin resistance, type 2 diabetes mellitus, cardiovascular disease, and liver steatosis-related conditions [1]. At the same time, the increasing prevalence of obesity is leading to a growing number of patients of reproductive age who are affected by these pathologies, which represent a challenge for obstetrics and gynecology specialists.

It is well known that maternal nutrition plays an essential role in fetal health [5,6]. The prevalence of maternal overweight/obesity is associated with an increased risk of congenital anomalies [5,7], such as skeletal, cardiac, neural tube and orofacial defects [5,8,9]. In addition, fetal and neonatal complications are more common, as well as postnatal metabolic complications in offspring of overweight/obese mothers, leading to an increased risk of obesity, insulin resistance and type 2 diabetes later in life [5,10,11,12,13].

Obesity is a pathologic condition mainly associated with an unhealthy lifestyle—high caloric intake and lack of physical activity—leading to morphologic changes in the structure of adipose tissue [3]. Adipose tissue is now regarded as an endocrine organ responsible for the hormonal secretion of adipokines, which are cytokines involved in various physiological processes [14,15,16,17]. Excessive body weight favors adipocyte hypertrophy and the occurrence of local cellular hypoxia, which together contribute to the development of chronic low-grade inflammation [3,18]. This leads to increased migration of immune cells, such as macrophages and lymphocytes, into pathologically remodeled adipose tissue due to increased adipocyte apoptosis promoted by hypoxia [3,19]. Increased infiltration of immune cells exposed to persistent hypoxia and increased oxidative stress disrupts the balance between secretion of anti- and proinflammatory cytokines [3,20,21].

Adipokines belong to the group of protein hormones and cytokines produced by adipocytes, immune cells, fibroblasts and other hormone-secreting cells derived from adipose tissue. Adipocytes release hundreds of signaling molecules that are responsible for the regulation of local and systemic cellular activities through their autocrine, paracrine and endocrine activity [22,23]. It has been established that adipokines play a key role in the regulation of many processes in the human body, such as glucose and lipid metabolism, insulin sensitivity, appetite, immune response and inflammation [24]. Due to their involvement in these processes, adipokines can be treated as potential targets for novel therapeutic strategies in numerous medical conditions [25].

Leptin and visfatin were selected for this study due to their established roles in metabolic and reproductive health, as well as their potential implications in obesity-related complications during pregnancy. Another reason for selecting this two adipocytokines was the existing data regarding the effect of the adipokines, leptin being a well investigated molecule and visfatin being recently discovered and data in literature being not that vast.

Leptin—a 16 kDa adipokine, composed of 167 amino acids, considered an adipocyte-derived satiety hormone—is elevated during pregnancy [1,26,27], and significantly higher in pregnant women with diabetes mellitus [27,28,29,30], suggesting leptin resistance. Outside pregnancy, leptin promotes energy intake and decreases food intake [27,31]. Leptin has also been shown to play a role in increasing insulin sensitivity [1,27], and in experimental studies using animal models (mice) lacking leptin, leptin treatment resulted in normalizing the serum insulin levels, improving glucose tolerance, all of which contribute to the correction of diabetes mellitus [27,32]. In the presence of obesity, the level of this adipokine is elevated (hyperleptinemia) but does not fulfill the role of reducing food intake or body mass index, indicating leptin resistance [27]. In addition, increasing the plasma level of leptin to a level similar to that observed in overweight and insulin-resistant individuals decreased glucose-mediated insulin secretion [1,27,33]. This suggests that leptin resistance in the presence of high leptin levels leads to a lack of action of this molecule, which favors the development of insulin resistance and obesity [27].

Visfatin, also known as Nicotinamide phosphoribosyl transferase (NAMPT), is a protein with enzymatic activity involved in the biosynthesis of Nicotinamide adenine dinucleotide (NAD+). NAD+ is a crucial coenzyme in various cellular processes including energy metabolism, DNA repair and cell signaling [1,34]. Research suggests that visfatin plays a role in several physiological and pathological conditions: cardiovascular and metabolic disorders- levels of visfatin are often elevated in people with obesity, type 2 diabetes and cardiovascular disease, it is thought to contribute to insulin resistance, inflammation and endothelial dysfunction. The exact mechanisms by which visfatin exerts its effects are still under investigation, but its involvement in NAD+ biosynthesis and various signaling pathways is a key research focus [14,34,35]. During pregnancy, visfatin levels undergo significant fluctuations, with potential implications for maternal and fetal health. This protein has been implicated in various aspects of pregnancy, including placental development, fetal growth and maternal metabolic adaptations. However, its precise role and underlying mechanisms remain unclear [14,36].

Flavonoids are a group of polyphenolic compounds found in plants, and they have been studied for their potential benefits on reproductive health. They have been found to have antioxidant and anti-inflammatory properties, and they may also play a role in regulating hormone levels, improving fertility outcomes, reducing risks of pregnancy complications and protection against oxidative stress [37].

The aim of the current study was to use an animal model to investigate the possible influence of obesity and adipokines on the gestational period, on the development of offspring, and to assess whether these changes are influenced by the administration of antioxidant agents and flavonoids.

## 2. Materials and Methods

The present study was performed using 5 groups of 7 female Wistar albino rats, after obtaining the approval of the Veterinary Sanitary Commission No. 321/02.08.2022 and the approval of the Ethics Committee of the University of Medicine and Pharmacy “Iuliu Hațieganu”, Cluj Napoca. We chose Wistar albino rats as an animal model for obesity research due to several factors including physiological similarities (they share many physiological similarities with humans, including metabolic pathways and hormonal regulation of energy balance, making them relevant for studying human obesity), genetic uniformity (Wistar albino rats are an inbred strain, meaning they have a high degree of genetic uniformity, this reduces variability in experimental results), well-characterized (this strain has been extensively studied and characterized, providing a wealth of baseline data for comparison), susceptibility to Diet-Induced Obesity (Wistar albino rats are susceptible to diet-induced obesity (DIO). They readily gain weight and develop obesity-related metabolic changes when fed a high-fat diet, mimicking the development of obesity in humans), availability (Wistar albino rats are widely available from commercial breeders, making them easily accessible for research purposes). The animals were housed in separate cages and kept under conditions of 65% humidity, 21 °C temperature, 12-h day/night cycle, with standardized food and water given ad libitum. A control group (Group 1) received a standard rat chow diet with 5% lipid diet. The other 4 groups were fed a high-fat diet (HFD) with 65% lipid content to induce obesity. The study groups were divided as follows:

- Group 1—control animals—normal weight pregnant females with an average weight of 300 g;
- Group 2—obese animals—pregnant females receiving HFD without supplement;
- Group 3—obese animals—pregnant females receiving HFD with flavonoids (Detralex);
- Group 4—obese animals—pregnant females receiving HFD with antioxidant supplement (Sel-E-Vit);
- Group 5—obese animals—pregnant females receiving HFD with administration of flavonoids (Rutin).

Supplements

Detralex: Detralex is a commercially available flavonoid medication containing 500 mg of purified flavonoid fraction per tablet, composed of 90% diosmin and 10% hesperidin. Each rat in Group 3 received 4.5 mg of Detralex per day via gavage feeding for 21 days (the whole length of gestational period). 

Sel-E-Vit: Sel-E-Vit is a commercially available antioxidant supplement containing Sodium Selenite 0.05 g and α-Tocopherol acetate 5 g per 100 mL solution. Each rat received 0.3 mL of Sel-E-Vit once every 2 weeks via subcutaneous injection for the whole gestational period.

Rutin: Rutin is a commercially available flavonoid supplement containing 500 mg of rutin per tablet. Each rat in Group 5 received 2 mg of Rutin per day via gavage feeding for 21 days (the whole length of gestational period).

Study medication was procured by the animal studies department following internal procedures in accordance with Good Laboratory Practices (GLP).

These supplements were chosen based on their known antioxidant and potential beneficial effects on metabolic and vascular health, which are often compromised in obesity. 

Study Timeline and Measurements

The study duration was 21 days, starting from the first day of gestation. The following measurements were taken at specific time points:

- day 15 of gestation: Venous blood was drawn from the pregnant females to determine plasma adipokine (leptin and visfatin) levels. This time point was chosen as it represents a critical period for placental development and fetal growth, and adipokine levels are known to fluctuate during gestation.
- days 18–22 of gestation: Ultrasound examination was performed to measure the thickness of adipose tissue in the abdominal wall. This period was chosen as it corresponds to the late stages of pregnancy when fat deposition is typically increased.
- offspring: After birth, 10 offspring from each group were selected for measurements. These included body weight, brain weight, and anthropometric measurements (head length, head width, spine length, width between shoulder blades, coxal bone length). Adipokine levels in the offspring’s brain tissue were also determined. Brain tissue samples were collected for electron microscopy analysis to identify any ultrastructural changes.

Measurement Methods

Leptin and Visfatin Levels: Plasma leptin and visfatin levels were determined using ELISA kits. Brain tissue adipokine levels were measured using ELISA kits. The acquisition of the study kits was realized by UMF “Iuliu Hațieganu” Cluj-Napoca through the purchasing departments in compliance with the internal rules in accordance with national laws and regulations

Body Weight: Body weight of both pregnant females and offspring was measured using a digital scale with an accuracy of 0.01 g.

Abdominal Wall Thickness: Abdominal wall thickness was measured using an ultrasound machine with a 7.5 MHz linear transducer. Measurements were taken at the level of the umbilicus in triplicate and averaged.

Electron Microscopy

For the brains structure study, frontal cortex samples of about 1 mm^3^, collected from the two control and three treatment groups, were prefixed 2 h in 2.7% glutaraldehyde (Electron Microscopy Sciences, Hatfield, PA, USA) in 0.1 M phosphate buffer, washed four times in the same buffer (1 h each) and postfixed 1.5 h in 1.5% OsO4 (Sigma-Aldrich, St. Louis, MO, USA) in 0.15 M phosphate buffer. Then they were dehydrated with acetone series (15–30 min each) and infiltrated with EMBed-812 epoxy resin (Electron Microscopy Sciences) series in acetone (1 h each). From blocks polymerized at 60 °C for 72 h sections of 60–80 nm were cut with a Diatome diamond knife on a Bromma 8800 ULTRATOME III ultramicrotome (LKB Produckter AB, Stockholm-Bromma, Sweden). The sections, collected on 300 mesh Cu electrolytic grids (Agar Scientific Ltd., Stansted, UK) covered with a formvar (Electron Microscopy Sciences) film, were contrasted 15 min with 13% uranyl acetate (Merck, Billerica, MA, USA) and 5 min with 2.8% lead citrate (Fluka AG, Buchs, Switzerland) and examined with a Jeol JEM-100CX II transmission electron microscope (Jeol, Tokyo, Japan) equipped with a Mega View G3 camera (emsis, Münster, Germany).

Statistical analysis

For all continuous variables, the mean and standard deviation were calculated. A two-sample *t*-test assuming equal variances was used to compare pairs of the means between each group receiving HFD plus supplement with the control group and, respectively, group receiving HFD only. Moreover, a comparison of means between the control group and group receiving HFD only was made for each parameter in order to assess the effect of HFD on mothers and offspring. Taking all of these into account, for both the mothers and the offspring, the variables coming from the high-fat diet plus supplements groups were compared with the corresponding variables of control groups and high-fat diet only groups, respectively. A significance threshold of 0.05 was used. The statistical software SPSS Statistics v29 was used for analyses.

## 3. Results

### 3.1. Comparative Analysis of Pregnant Groups

In terms of body weight, we compared the groups included and we observed that females in group 2 had significantly increased weight compared to those in group 1. The same pattern was observed when group 3 was compared to group 1, but no statistical significance was obtained when comparing group 3 (High Fat Diet- HFD+Detralex) with group 2 (animals that received only HFD). Body weight of animals in group 4 (HFD+Sel-E-Vit) was also compared with body weight of animals in group 1 and 2, and an increased weight in group 4 compared to group 1, and no difference between groups 4 and 2. The same comparisons were made also between groups 5 (HFG+Rutin), 1 and 2 and we observed that females in group 5 gained significantly more weight than both group 1 and group 2 (Table 1). We also have compared the weight variation between the study groups. The HFD (high-fat diet) groups (2, 3, 4, and 5) all show a statistically significant increase in weight compared to the control group (Group 1). The group receiving HFD+Rutin (Group 5) gained significantly more weight than even the HFD-only group (Group 2). This suggests that Rutin may have had an unexpected effect on weight gain when combined with an HFD. There were no statistically significant differences in weight variation between the other HFD groups (3 and 4) compared to the HFD-only group (2).

Another parameter that was investigated in the pregnant groups is represented by the abdominal wall thickness. Females in group 2 had a significantly thicker abdominal wall than those in group 1. The same difference was also observed when comparing females in group 3, 4 and 5 with those in group 1, but no statistical significance was obtained when comparing those groups with the pregnant females in group 2 (Table 1).

After being generally accepted that adipose tissue is an important endocrine organ another aim of our study was to investigate if there are differences between the plasma levels of two of the cytokines produced by the adipocytes (leptin and visfatin). In terms of plasma leptin concentration, females in group 2 had a significantly higher leptin level than those in group 1. The leptin values of female rats in group 3 were compared to those of females in groups 2 and 1 with no statistically significant difference obtained. When comparing the levels of this adipocytokine from group 4 the result was that females in group 4 had significantly higher levels than those in group 1 with no statistically significant difference between females in group 4 and group 2. Leptin levels of the females in group 5 did not differ significantly from those in females in group 1 (control group), but it was found that mean levels were lower in group 5 (animals that received HFD+Rutin) when compared with those in group 2 (animals that received only HFD) (Table 1).

In terms of plasma visfatin concentration of the female groups of rats included in our study, there were no statistically significant differences when comparing levels of visfatin between groups excepting the finding of a lower level of this molecule in group 5 compared to the values the control group (Table 1).

We have also used Pearson correlation coefficients to analyze the existence of correlations between the levels of adipocytokines (leptin and visfatin) and body weight, body weight gain, and abdominal wall thickness.

In terms of analyzing the correlations between plasma leptin levels the results show a strong positive correlation with body weight at 6 weeks across all groups, particularly in Group 3 (0.88). This suggests that as body weight increases, plasma leptin levels tend to increase as well (Table 2). Variation of body weight also exhibits strong positive correlations with plasma leptin levels, especially in Group 3 (0.87). This indicates that changes in body weight are associated with changes in plasma leptin levels (Table 2). Abdominal wall thickness shows moderate positive correlations with plasma leptin levels, particularly in Group 3 (0.64). This suggests that as abdominal wall thickness increases, plasma leptin levels tend to increase, but the relationship is not as strong as with body weight (Table 2).

Regarding the correlation of Visfatin plasma levels the results in Table 3 show strong positive correlations with body weight at 6 weeks against all groups, with the strongest correlation in Group 3 (0.93). This suggests that as body weight increases, plasma visfatin levels tend to increase as well. Variation of body weight exhibits strong correlations with plasma visfatin levels, with the strongest correlation in Group 3 (0.96). This indicates that changes in body weight have a important association with changes in plasma visfatin levels. Abdominal wall thickness shows strong correlations with plasma visfatin levels across all groups, with the strongest correlation in Group 5 (0.97). This suggests that there is a linear relationship between abdominal wall thickness and plasma visfatin levels.

### 3.2. Comparative Analysis of Offspring

#### 3.2.1. Body Weight

Regarding the effects of maternal obesity on offspring, our study investigated the occurrence of differences in body weight of descendants from the study groups. The offspring from the obesogenic diet group females (Group 2) had a significantly higher body mass compared to the ones resulted from the control group (Group 1). Offspring resulted from females of Group 3 (HFD+ Detralex) had statistically significant lower body weight values than the ones from females of the control group (Group 1) and, obviously, than the offspring from the females of the HFD group (Group 2). Comparing the offspring from group 4 (HFD+ Sel-E-Vit) with the ones from group 1 and 2 our study showed that descendants from group 4 had statistically significant lower values of body weight than the ones resulting from the control group and from females in group 2. The same pattern was also found when comparing offspring of female rats from group 5 (HFD+Rutin) with the ones from the control group and group 2 with statistically significant lower values in group 5 compared to the other 2 groups (Table 4).

#### 3.2.2. Brain Weight

Another parameter investigated in our study was the brain weight of offspring resulted. Brain weight of descendants from each group of obese female rats was compared with the brain weight of descendants in group 1 and then the brain weight of offspring of obese female rats that also received an antioxidant or flavonoid compound were compared also to the values obtained from offspring in group 2 (HFD). A statistically significant difference was found when comparing the brain weights of offspring from groups 2, 3 and 5 to group 1, with higher values in group 2 compared to the ones in group 1 and lower levels in group 3 and 5 compared to offspring in group 1. No difference was found in brain weights of offspring in group 4 (HFD+Sel-E-Vit) and group 1. Compared to group 2, there was a statistically significant difference, with lower values of brain weigh found in groups 3, 4 and 5 (Table 4).

#### 3.2.3. Head Length

During our study we also investigated the occurrence of any anthropometric differences between the offspring resulted from the study groups. In terms of head length, the head length of offspring from HFD group (Group 2) was significantly longer than the control group (Group 1). Data collected from offspring in groups 3, 4 and 5 showed statistically significant lower values of head length compared to HFD group and control group (Table 4).

#### 3.2.4. Head Width

Another anthropometric value investigated during our study was head width of offspring. The values obtained from each group of descendants resulting from obese female rats were compared with the values obtained from the control group. Also, values obtained from offspring of females that received an antioxidant or a flavonoid compound aside from the obesogenic diet were also compared to the values found in offspring of females in group 2 (HFD). There was a statistically significant difference, with higher values in offspring of females in group 2 compare to the ones obtained from the descendants of females in the control group. Values obtained from descendants in groups 3, 4 and 5 were compared to the ones obtained from group 2 and group 1. The statistical analysis showed lower values of head width in groups 3, 4 and 5 than the head width of offspring in group 2 and group 1.

#### 3.2.5. Spine Length

In terms of anthropometric measurements another parameter investigated in our study was the spine length. Comparing the spine length of offspring of females from group 2 with the ones of females in the control group (Group 1) the values obtained were significantly higher in offspring of HFD (Groups 2) than in the control group (Group 1).

Comparing the spine length of descendants in the rest of the groups 3, 4, 5 with the values obtained in group 2 and group 1 our results showed statistically significant lower values in the offspring resulted from the females that received an antioxidant or flavonoid compound along with the HFD (Table 4).

#### 3.2.6. Width Between Shoulder Blades

Another anthropometric parameter investigated was the width between the shoulder blades of the offspring. Comparing the values obtained from the descendants of females in HFD group with the ones from the control group, the vales showed statistically significant higher values in the offspring in group 2. Comparing the same values with the measurements obtained from the descendants of females in groups 3, 4 and 5, our results show lover values of the width between shoulder blades in offspring of females in groups 3, 4 and 5 compared to both group 2 and group 1 (Table 4).

#### 3.2.7. Coxal Bone Length

The last anthropometric parameter included is the length of the coxal bone. Our results show statistically significant higher values in offspring from HFD females (Group 2) compared to the values obtained from offspring of the control group. The same comparison was made between the coxal bone length of offspring of females in groups 3, 4 and 5, results showing statistically significant lower values of the coxal bone length in offspring from females that received Detralex, Sel-E-Vit and Rutin along with HFD compare to the values obtained from the control group and obviously with the group that received only HFD alone (Table 4).

#### 3.2.8. Brain Levels of Adipocytokines (Leptin and Visfatin)

During our study we have also investigated the levels of the two adipocytokines in the brain of the offspring. The results show there was no statistically significant difference in brain leptin levels between the offspring of the control group and those of the high-fat diet (HFD) group. Additionally, the offspring of the HFD group that received rutin also showed no significant difference in brain leptin levels compared to the control group. The levels of brain visfatin in the offspring of the HFD group that received Sel-E-Vit were significantly lower compared to both the control group and the HFD-only group. There were no significant differences in brain visfatin levels between the offspring of the control group and those of the HFD group, or those of the HFD groups that received Detralex or rutin (Table 2).

#### 3.2.9. Brain Structure Modifications

**Figure 1:**
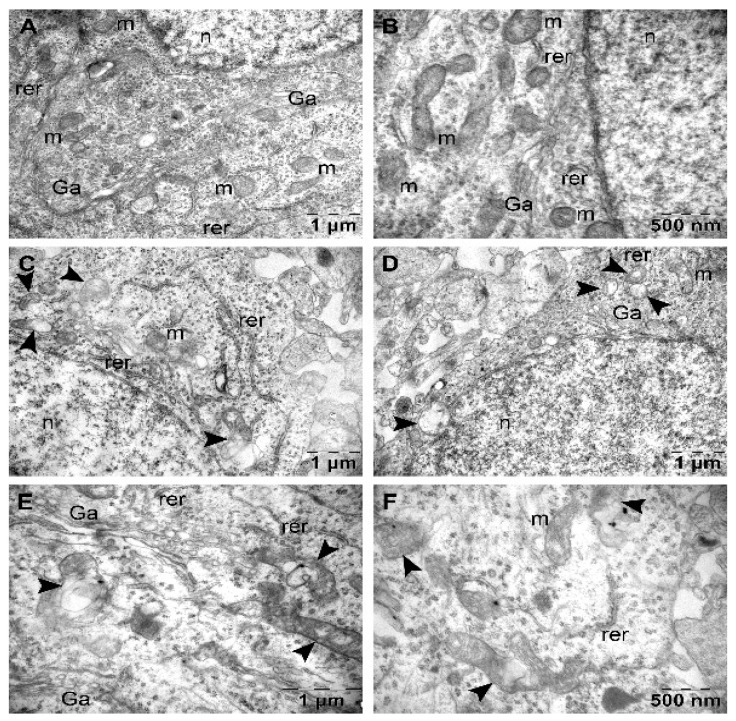
Neurons with normal ultrastructural aspect in the negative control group (**A**,**B**), and with mitochondrial alterations in the positive control group (**C**–**F**). Ga, Golgi apparatus; m, mitochondrion (normal); n, nucleus; rer, rough endoplasmic reticulum; arrowheads, altered mitochondria.

In the negative control group, TEM examination revealed the normal ultrastructure of the neurons (Figure 1A,B) and blood capillaries. Neurons contained large, round, euchromatic nucleus, many profiles of rough endoplasmic reticulum, 1–3 Golgi complexes and many round-oval (Figure 1A) or even elongated (Figure 1B) mitochondria.

In the positive control group (DBL), neurons showed slightly irregular contour and extensive ultrastructural changes of mitochondria (Figure 1C–F). Some of such altered mitochondria preserved the round-oval shape, but their matrix was either partially electron-transparent and contained modified (vesicular) cristae (Figure 1C,D), or entirely electron-transparent, without visible cristae (Figure 1D). Many other abnormal mitochondria were polymorphous, with distinct regions of rarefied matrix and/or vesicular, large cristae (Figure 1C,E,F). The endoplasmic reticulum and Golgi apparatus showed no changes.

**Figure 2:**
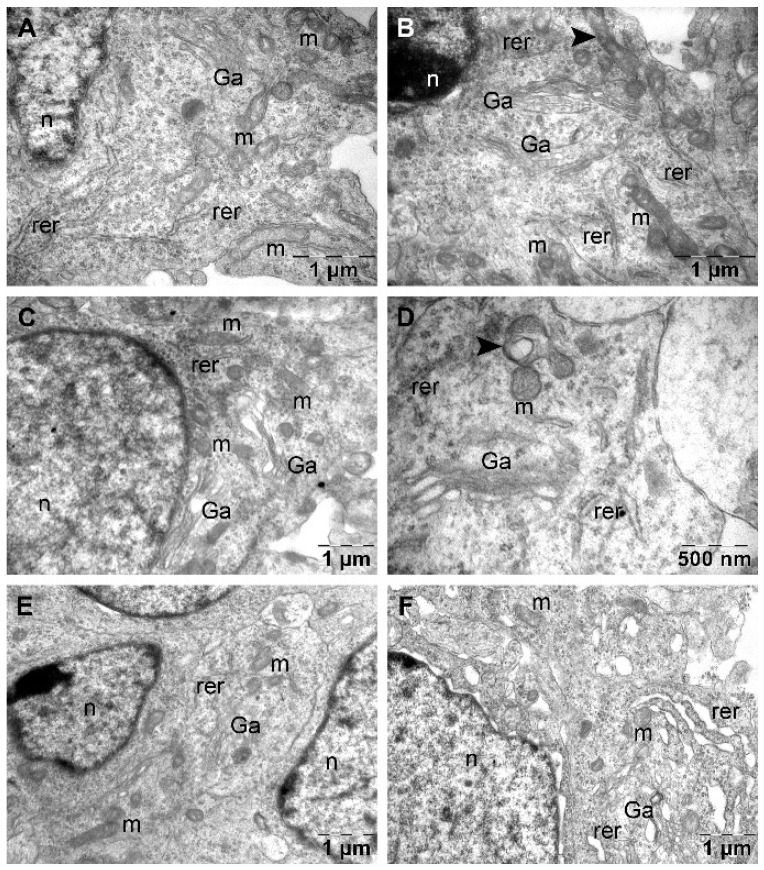
Ultrastructural aspects of neurons in the treatment groups: normal neuron in DBL-Detralex group (**A**) and minor mitochondrial change (**B**): normal neuron in DBL-Selevit group (**C**) and accentuated mitochondrial change (**D**); normal neuron in DBL-Rutin group (**E**) and expansion of perinuclear space and rough endoplasmic reticulum (**F**). Ga, Golgi apparatus; m, mitochondrion (normal); n, nucleus; rer, rough endoplasmic reticulum; arrowhead, altered mitochondrion.

In the DBL-Detralex group, all the studied neurons had normal ultrastructural aspect (Figure 2A,B). Only rare mitochondria contained vesicular cristae in a normal, granular matrix (Figure 1B).

In the DBL-Sel-E-Vit group, most of the studied neurons had normal ultrastructural aspect (Figure 2C). In some neurons 1–2 polymorphous mitochondria were found, that also contained 1–2 large vesicular cristae in a normal matrix (Figure 2D).

In the DBL-Rutin group, some neurons displayed normal aspect (Figure 2E), while in many others expansion of the perinuclear space and of rough endoplasmic reticulum was noted as important ultrastructural changes were noted (Figure 2F).

Apart from the presented ultrastructural aspects, and not shown into the selected figures, we noted dilated Golgi apparatus in some neurons of the DBL-Selevit group, lipid droplets in the cytoplasm of endothelial cells from the DBL-Detralex group, and constant presence of large multivesicular bodies in the endothelial cells from the first four groups (excepting the DBL-Rutin group).

## 4. Discussion

The current study sought to bring to the forefront the effects of gestational obesity on both pregnant females, the levels of molecules recently discovered to be secreted by adipose tissue (adipocytokines), and the product of conception. Due to the limited number of studies investigating the existence of methods by which overweight as well as adipocytokine levels could be controlled during the gestational period, the possible effects of the administration of antioxidant or flavonoid compounds were investigated, and their effect on the resulting offspring was also followed.

First, it was observed that the high lipid diet induced significant weight gain in females, with a statistically significant difference between the weights of females in the control group and those given the high lipid diet. This difference was also maintained between the groups of female rats given a flavonoid compound (Rutin and Detralex) in addition to the lipid-rich diet and between the control group and the group given an antioxidant compound (Sel-E-Vit). The weights of the animals in the group given the flavonoid compound Rutin in addition to the lipid-rich diet were significantly higher than the weights of the animals in the group given only the HFD. A study published in the British Journal of Nutrition titled “Obesity induced by cafeteria feeding and pregnancy outcome in the rat” also used Wistar albino rats as an obesity model. This study investigated the impact of maternal obesity induced by a cafeteria diet (a highly palatable, energy-dense diet) on pregnancy outcomes in rats. The researchers found that maternal obesity negatively affected pregnancy outcomes, including increased risk of fetal abnormalities and impaired placental function. The weight variation data shows the impact of different diet interventions on the weight of pregnant rats. All high-fat diet (HFD) groups (2, 3, 4, and 5) experienced significant weight gain compared to the control group (Group 1), as expected. Interestingly, the group receiving HFD + Rutin (Group 5) gained significantly more weight than even the HFD-only group (Group 2), suggesting an unexpected interaction effect between Rutin (flavonoid compound) and HFD on weight gain. There were no significant differences in weight variation between the other HFD groups (3 and 4) compared to the HFD-only group (2), indicating that Detralex and Sel-E-Vit did not significantly influence the weight gain caused by HFD. These findings highlight the importance of considering the potential influence of flavonoids on weight gain, particularly in the context of HFD consumption. Further research is needed to understand the mechanisms underlying the observed interaction between Rutin and HFD and its implications for weight management during pregnancy [27].

The presence of obesity was also assessed by measuring abdominal wall thickness. A statistically significant difference was observed between the control group and the other groups of animals with higher abdominal wall thickness. No significant differences in abdominal wall thickness were found between the group of females given only the high-fat diet and the groups given antioxidant or flavonoid compounds. These data are consistent with data in the literature showing a positive relationship in humans between the amount of dietary energy from dietary fat and the proportion of overweight population (in epidemiologic studies), and in clinical studies between dietary fat level and body weight gain, and between dietary fat reduction and weight loss [38,39,40]. These associations have also been shown in experimental studies [38,41] in which higher dietary fat models lead to greater obesity, suggesting that an important factor in energy balance is the fat content of the diet. In general, diets containing more than more than 30% of total energy as fat lead to obesity [38]. A weakness of this study is the limited number of animals included in this study. However, the study’s strength lies in its comprehensive approach, investigating the effects of maternal obesity on offspring, including anthropometric measurements, brain weight, and ultrastructural changes in the brain. The study also investigated the potential mitigating effects of antioxidant and flavonoid compounds on these parameters.

Another parameter investigated by the current study, and which represents one of the strong points, was the level of the adipokines leptin and visfatin, as these molecules are known to be produced by adipose tissue and may influence the course of pregnancy by the development of pathologies associated with this period, being recently discovered and the knowledge about their mechanism of action and their effects is reduce. The results of the current study could contribute to improving the knowledge about these molecules. Leptin is considered an important metabolic hormone, influencing insulin secretion, glucose utilization, glycogen synthesis and fatty acid metabolism. Leptin is released into the circulation by adipose tissue in proportion to the amount of lipid stores and acts at hypothalamic receptors, reducing food intake and increasing energy expenditure [41,42]. The expression and action of leptin are altered in metabolic disorders associated with insulin resistance, such as obesity and gestational diabetes mellitus [41,43]. In addition, leptin plays a key role in immune response and T cell activation [41,44,45]. Visfatin is predominantly secreted by adipose tissue, but is also synthesized in other tissues such as skeletal muscle, liver, immune cells, cardiomyocytes and brain [36]. Visfatin exhibits intrinsic NAMPT activity, with its intracellular form (iNAMPT) regulating intracellular levels of nicotinamide adenine dinucleotide (NAD) and its extracellular form acting as a cytokine in response to inflammatory stimuli or cellular stress [46]. It is involved in the synthesis of NAD and has been associated with the development of obesity, insulin secretion, lipid profile and inflammation, among others. Visfatin was originally reported as an insulin mimetic adipokine that binds to and activates the insulin receptor promotes adipogenesis, stimulates glucose uptake in vitro, and exerts glucose-lowering effects in mice in vivo [36,46]. The results of our study are in agreement with literature data and show that adipokine levels are influenced by the degree of obesity showing significantly higher levels of leptin in the group of female rats given a high-fat diet compared with the levels of this molecule in the control group. The data of our study also show that leptin levels are not influenced by the administration of bioflavonoid compounds (Detralex) or antioxidants, but they were significantly higher in the group of animals that received the natural flavonoid (Rutin) in addition to the lipid-rich diet. The levels of the second molecule investigated in the study, visfatin, were numerically lower in the obese groups compared to the control group, with statistical significance being reached only in the group of animals given natural flavonoid (Rutin) in combination with the high lipid diet. This finding is not in agreement with the existing data in the literature, which show that the levels of this molecule are influenced by the presence of obesity, that visfatin values increase during the gestational period, and in pregnancies with pathologies associated with the gestation period such as gestational diabetes mellitus [36]. The results observed strong positive correlations between plasma leptin and physiological parameters, especially body weight and body weight variation. These findings are consistent with existing literature, which shows that leptin levels are influenced by the degree of obesity [36,41,42]. Leptin, an adipokine produced by adipose tissue, is known to play a crucial role in regulating energy balance, metabolism, and various physiological processes. Higher leptin levels are typically associated with increased body weight and adiposity, as leptin is released into circulation in proportion to the amount of lipid stores. Leptin’s actions at hypothalamic receptors are thought to reduce food intake and increase energy expenditure, contributing to weight regulation [41,42]. Our study found strong correlations between plasma visfatin and physiological parameters. 

This finding contrasts with existing literature, which suggests that visfatin levels are influenced by the presence of obesity and may increase during gestation and in pregnancies with associated pathologies. While visfatin was initially reported as an insulin mimetic adipokine with potential implications for metabolic health, its precise role and mechanisms of action remain unclear [46,47]. Further investigation is needed to fully understand the complex interplay between visfatin, obesity, and related physiological parameters.

Also in our study we analyzed the effects of maternal obesity on offspring, as well as the existence of effects of the administered compounds on the weight of offspring, their brain weight, the existence of changes in the brain detected by electron microscopy, anthropometric changes (length and width of head, vertebral column length, width between shoulder blades, coxal bone length) and also modifications of the levels of the adipocytokines in offspring, with data in the literature emphasizing that obesity during gestation has both immediate and long-lasting effects on the fetus [47]. These data bring an added value to the study, as there are few specialized studies addressing both the offspring and possible ways to ameliorate the effects of this pathology, which is increasingly common nowadays.

The results of the study in terms of the weight of the resulting offspring are in agreement with existing data that maternal obesity during pregnancy influences birth weight, the offspring of the females in the high lipid diet group had significantly higher weights than the offspring of the control group [2,47,48]. The other groups of animals receiving a flavonoid or antioxidant compound in addition to the high-fat diet had significantly lower birth weights than the offspring from the group of females on the high-fat diet alone and even the offspring from the control group. The resulting offspring brain weights were significantly higher in the descendants of the group of females receiving only the high lipid diet, but this difference was not maintained in the other groups. The brain weights of offspring were significantly lower in the ones resulting from the female groups given a flavonoid, both compared to those from the high lipid diet group and to the brain weights of offspring from the control group. In the offspring from the group of females receiving an antioxidant compound associated with the obesogenic diet, brain weights were significantly lower than in the offspring from the group of females receiving only the obesogenic diet, but brain weights were similar to those of the offspring from the control group. As regards anthropometric measurements (head length and width, spine length, width between shoulder blades, coxal bone length) carried out on offspring from the 5 groups of animals, the data obtained showed a statistically significant difference between offspring from females receiving the high-fat diet and offspring from the other groups, all the dimensions evaluated being larger in the offspring from the group of females receiving the HFD, results which are in agreement with the literature, maternal obesity being a predisposing factor for the development of large for gestational age or macrosomic fetuses [2,4,7,8]. A finding of the current study is that the offspring of female rats from the groups that received a flavonoid compound associated with the lipid diet had significantly smaller sizes of all evaluated parameters compared to both offsprings of females from the group that received the lipid diet, but more interestingly also to offspring from control group females. The same differences in the body weight and brain weights of the descendants were found between the groups in which the females received flavonoid compounds and those in which the female rats received 5% lipid diet and HFD. There was no significant difference in brain leptin levels between the offspring of the control group and the HFD group, or between the offspring of the HFD group that received antioxidants or flavonoid compounds and the control group. This finding is consistent with previous research indicating that brain leptin levels are primarily regulated by energy balance and are not significantly affected by maternal diet or nutritional interventions [27,35]. However, it is important to note that leptin signaling in the brain is complex and involves multiple factors, including leptin receptor expression and sensitivity, as well as the interaction of leptin with other hormones and neuropeptides [27]. Therefore, further research is needed to fully understand the impact of maternal HFD and flavonoid supplementation on leptin signaling in the offspring’s brains.

The levels of brain visfatin in the offspring of the HFD group that received antioxidant compound were significantly lower compared to both the control group and the HFD-only group. This finding suggests that antioxidant supplementation may have a protective effect against the potential negative effects of maternal HFD on brain visfatin levels in the offspring. Visfatin, also known as nicotinamide phosphoribosyl transferase (NAMPT), is an enzyme involved in the biosynthesis of nicotinamide adenine dinucleotide (NAD+), a crucial coenzyme in various cellular processes, including energy metabolism, DNA repair, and cell signaling. Studies have shown that visfatin plays a role in regulating insulin secretion, glucose homeostasis, and inflammation. Dysregulation of visfatin has been implicated in various metabolic disorders, including obesity, type 2 diabetes, and cardiovascular disease [34,35]. The observed decrease in brain visfatin levels in the antioxidant supplemented group may be attributed to the antioxidant and anti-inflammatory properties of Sel-E-Vit. Oxidative stress and inflammation are known to contribute to the development of metabolic disorders and may also affect visfatin expression and activity. By reducing oxidative stress and inflammation, antioxidant supplementation may help maintain normal visfatin levels in the offspring’s brains, potentially protecting against the adverse effects of maternal HFD. There were no significant differences in brain visfatin levels between the offspring of the control group and those of the HFD group, or those of the HFD groups that received flavonoid compounds. The lack of significant differences in brain visfatin levels between these groups suggests that maternal HFD and flavonoid supplementation did not significantly affect visfatin levels in the offspring’s brains. This finding is consistent with previous research indicating that brain visfatin levels are relatively stable and are not significantly affected by maternal diet or nutritional interventions [35,36]. Considering the results of the brain evaluations of the offspring in the study groups in the control group normal aspects of cerebral substance were recorded, in contrast to the group receiving the high lipid diet where abnormalities of both neurons and mitochondria were observed. Significant changes, however, were observed in the group of offspring derived from females receiving the flavonoid supplement. From the above data we can say that the results of the study in terms of the effects of obesity on offspring are in agreement with existing data in the literature, but the results of our study shed light on possible adverse effects of flavonoids on the developing conception product. Flavonoids are a subgroup of polyphenols with a common C6-C3-C6 common chemical structure [37,48,49]. They are naturally present in many plants and are incorporated into our diets, e.g., as components of fruits, vegetables and beverages such as coffee tea and fruit juices [37,49,50]. Dietary flavonoids have been proposed to have a number of potential health benefits, including the prevention and even treatment of several chronic diseases such as cancer, cardiovascular and neurodegenerative diseases [47,48,51], which is why their use in the general population has been increasing. Herbal supplements are intended for the general population, but certain population subgroups may be more sensitive, such as pregnant women. There are a limited number of studies on the effects of these compounds during gestational period, but the results of our study reinforce some findings that some flavonoids may cause some adverse developmental effects such as adverse effects on reproductive development, nervous system development, genetic toxicity or growth retardation [37,48]. Despite indications that this group of compounds might exert adverse effects on developmental processes, there is a clear lack of data, for example, there are almost no studies on osteogenesis or on the development of the cardiovascular system. In addition, there has been only one in vivo neurodevelopmental study in vivo for a flavanone, but not for other flavonoids. In combination with the lack of data for individual flavonoids, this paucity of data for more specific developmental effects leaves high uncertainty for characterizing the hazard and risk of flavonoids. In addition, very few studies have investigated the molecular mechanisms of flavonoid toxicity [37,48,52], there is an urgent need for more mechanism-based evaluations of flavonoid mechanism of action to establish safety margins for single flavonoid intake during pregnancy.

The main limitation of this study is the small sample size (7 rats per group). Being a pilot study, the dimension of the groups was not decided based on a sample size calculation. An arbitrary of 7 females for each female group was decided. The number of 10 animals for offspring groups was decided taking into account the smallest number of offspring resulting from mother groups. Thus, for the groups in which resulted more than 10 offspring, a number of 10 was randomly selected by the investigator. This may limit the generalizability of the findings and increase the risk of type II error (failing to detect a real effect). However, despite this limitation, the results can still be considered valid and an important signal due to the following factors:

- rigorous methodology: the study followed a well-defined protocol with standardized procedures for animal care, diet, supplement administration, and measurements.
- significant differences: despite the small sample size, statistically significant differences were observed between the groups for many of the measured parameters, suggesting that the effects are robust.
- consistency with previous research: the findings regarding the effects of maternal obesity on offspring are consistent with existing data in the literature, supporting the validity of the results.
- detailed analysis: The study included a comprehensive analysis of various parameters, including adipokine levels, anthropometric measurements, and brain ultrastructure, providing a more complete picture of the effects of maternal obesity and flavonoid supplementation.

To further strengthen the validity of the findings and address the limitation of the small sample size, future studies should be conducted with larger cohorts and potentially different animal models. Additionally, further investigation into the specific mechanisms underlying the observed effects, particularly the potential adverse effects of flavonoids on offspring development, is warranted.

## 5. Conclusions

The results of this experimental study reinforce what is already known about the effects of obesity on the gestation period and offspring. The high-fat diet leads to increased weight gain in females and to the occurrence of increased size offspring compared to the control group, as it is already known that maternal obesity leads to the appearance of macrosomia. Obesity also produces changes in the offspring’s cerebral tissue, which may have long-term consequences, but further studies on brain changes and their development are needed. The levels of molecules produced by adipose tissue (adipocytokines) increase in direct proportion to the degree of obesity and are involved in the development of pregnancy-related pathologies and the effects of obesity on offspring.

At the same time, the current study highlights the existence of possible adverse effects of flavonoid compounds on the development of pregnancy and offspring, opening the way for future studies on the benefits and risks of using these compounds during gestational period.

## Figures and Tables

**Table 1:** Comparative analysis of female groups.

	Mean	SD	Mean	SD	*p*
Weight at end of study					
Group 1 vs. Group 2	222.86	11.47	456.71	31.71	<0.001
Group 3 vs. Group 1	454.29	32.69	222.86	11.47	<0.001
Group 3 vs. Group 2	454.29	32.69	456.71	31.71	NS *
Group 4 vs. Group 1	459.14	15.25	222.86	11.47	<0.001
Group 4 vs. Group 2	459.14	15.25	456.71	31.71	NS
Group 5 vs. Group 1	493.57	16.97	222.86	11.47	<0.001
Group 5 vs. Group 2	493.57	16.97	456.71	31.71	<0.01
Weight variation					
Group 1 vs. Group 2	0.0	0.0	241.29	32.55	<0.001
Group 3 vs. Group 1	237.29	32.29	0.0	0.0	<0.001
Group 3 vs. Group 2	237.29	32.29	241.29	32.55	NS
Group 4 vs. Group 1	236.0	17.40	0.0	0.0	<0.001
Group 4 vs. Group 2	236.0	17.40	241.29	32.55	NS
Group 5 vs. Group 1	279.71	15.28	0.0	0.0	<0.001
Group 5 vs. Group 2	279.71	15.28	241.29	32.55	<0.01
Abdominal wall thickness					
Group 1 vs. Group 2	1.30	0.29	2.49	0.56	<0.001
Group 3 vs. Group 1	2.19	0.35	1.30	0.29	<0.001
Group 3 vs. Group 2	2.19	0.35	2.49	0.56	NS
Group 4 vs. Group 1	2.39	0.20	1.30	0.29	<0.001
Group 4 vs. Group 2	2.39	0.20	2.49	0.56	NS
Group 5 vs. Group 1	2.46	0.15	1.30	0.29	<0.001
Group 5 vs. Group 2	2.46	0.15	2.49	0.56	NS
Plasma leptin					
Group 1 vs. Group 2	1.12	0.57	2.01	0.34	<0.01
Group 3 vs. Group 1	1.68	1.12	1.12	0.57	NS
Group 3 vs. Group 2	1.68	1.12	2.01	0.34	NS
Group 4 vs. Group 1	2.48	0.93	1.12	0.57	<0.05
Group 4 vs. Group 2	2.48	0.93	2.01	0.34	NS
Plasma visfatin					
Group 1 vs. Group 2	3.57	0.60	3.38	1.68	NS
Group 3 vs. Group 1	2.90	1.18	3.57	0.60	NS
Group 3 vs. Group 2	2.90	1.18	3.38	1.68	NS
Group 4 vs. Group 1	3.29	0.48	3.57	0.60	NS
Group 4 vs. Group 2	3.29	0.48	3.38	1.68	NS
Group 5 vs. Group 1	2.53	0.56	3.57	0.60	<0.05
Group 5 vs. Group 2	2.53	0.56	3.38	1.68	NS

* NS-Statistically not significant.

**Table 2:** Pearson correlation coefficients between body weight at 6 weeks, variation of body weight, and abdominal wall thickness with plasma levels of leptin for female groups.

	Leptin				
	Group 1	Group 2	Group 3	Group 4	Group 5
Body weight	0.88	0.78	0.88	0.97	0.93
Body weight variation	*	0.85	0.83	0.76	0.80
Abdominal wall thickness	0.84	0.72	0.89	0.94	0.87

* For group 1 the body weight variation in 6 weeks was zero and for this reason the correlation coefficient cannot be computed.

**Table 3:** Pearson correlation coefficients between body weight at 6 weeks, variation of body weight, and abdominal wall thickness with plasma levels of visfatin for female groups.

	Visfatin				
	Group 1	Group 2	Group 3	Group 4	Group 5
Body weight	0.90	0.92	0.93	0.84	0.89
Body weight variation	*	0.87	0.96	0.80	0.78
Abdominal wall thickness	0.80	0.88	0.84	0.86	0.97

* For group 1 the body weight variation in 6 weeks was zero and for this reason the correlation coefficient cannot be computed.

**Table 4:** Comparative analysis of the offspring groups classified based on the female groups.

	Mean	SD	Mean	SD	*p*
Weight					
Group 1 vs. Group 2	8.63	0.68	12.38	0.76	<0.001
Group 3 vs. Group 1	6.03	0.47	8.63	0.68	<0.001
Group 3 vs. Group 2	6.03	0.47	12.38	0.76	<0.001
Group 4 vs. Group 1	6.80	0.39	8.63	0.68	<0.001
Group 4 vs. Group 2	6.80	0.39	12.38	0.76	<0.001
Group 5 vs. Group 1	5.75	0.22	8.63	0.68	<0.001
Group 5 vs. Group 2	5.75	0.22	12.38	0.76	<0.001
Brain weight					
Group 1 vs. Group 2	0.31	0.04	0.49	0.05	<0.001
Group 3 vs. Group 1	0.25	0.03	0.31	0.04	<0.01
Group 3 vs. Group 2	0.25	0.03	0.49	0.05	<0.001
Group 4 vs. Group 1	0.31	0.04	0.31	0.04	NS *
Group 4 vs. Group 2	0.31	0.04	0.49	0.05	<0.001
Group 5 vs. Group 1	0.25	0.03	0.31	0.04	<0.001
Group 5 vs. Group 2	0.25	0.03	0.49	0.05	<0.001
Head length					
Group 1 vs. Group 2	18.13	0.70	21.27	0.53	<0.001
Group 3 vs. Group 1	16.33	0.42	18.13	0.70	<0.001
Group 3 vs. Group 2	16.33	0.42	21.27	0.53	<0.001
Group 4 vs. Group 1	16.87	0.42	18.13	0.70	<0.001
Group 4 vs. Group 2	16.87	0.42	21.27	0.53	<0.001
Group 5 vs. Group 1	15.64	0.39	18.13	0.70	<0.001
Group 5 vs. Group 2	15.64	0.39	21.27	0.53	<0.001
Head width					
Group 1 vs. Group 2	11.82	0.77	13.45	0.33	<0.001
Group 3 vs. Group 1	11.39	0.42	11.82	0.77	NS
Group 3 vs. Group 2	11.39	0.42	13.45	0.33	<0.001
Group 4 vs. Group 1	12.34	0.68	11.82	0.77	NS
Group 4 vs. Group 2	12.34	0.68	13.45	0.33	<0.001
Group 5 vs. Group 1	10.96	0.14	11.82	0.77	<0.01
Group 5 vs. Group 2	10.96	0.14	13.45	0.33	<0.001
Spine length					
Group 1 vs. Group 2	28.03	0.77	38.37	0.63	<0.001
Group 3 vs. Group 1	25.43	0.50	28.03	0.77	<0.01
Group 3 vs. Group 2	25.43	0.50	38.37	0.63	<0.01
Group 4 vs. Group 1	26.70	0.77	28.03	0.77	<0.001
Group 4 vs. Group 2	26.70	0.77	38.37	0.63	<0.001
Group 5 vs. Group 1	25.26	0.48	28.03	0.77	<0.001
Group 5 vs. Group 2	25.26	0.48	38.37	0.63	<0.001
Width between shoulder blades					
Group 1 vs. Group 2	13.01	0.61	15.17	0.32	<0.001
Group 3 vs. Group 1	11.83	0.77	13.01	0.61	<0.001
Group 3 vs. Group 2	11.83	0.77	15.17	0.32	<0.001
Group 4 vs. Group 1	12.25	0.67	13.01	0.61	<0.01
Group 4 vs. Group 2	12.25	0.67	15.17	0.32	<0.001
Group 5 vs. Group 1	11.24	0.70	13.01	0.61	<0.001
Group 5 vs. Group 2	11.24	0.70	15.17	0.32	<0.001
Coxal bone length					
Group 1 vs. Group 2	15.86	0.99	16.30	0.36	NS
Group 3 vs. Group 1	14.07	0.51	15.86	0.99	<0.001
Group 3 vs. Group 2	14.07	0.51	16.30	0.36	<0.001
Group 4 vs. Group 1	14.88	0.82	15.86	0.99	<0.05
Group 4 vs. Group 2	14.88	0.82	16.30	0.36	<0.001
Group 5 vs. Group 1	13.81	0.51	15.86	0.99	<0.001
Group 5 vs. Group 2	13.81	0.51	16.30	0.36	<0.001
Brain leptin					
Group 1 vs. Group 2	0.27	0.08	0.29	0.09	NS
Group 3 vs. Group 1	0.30	0.10	0.27	0.08	NS
Group 3 vs. Group 2	0.30	0.10	0.29	0.09	NS
Group 4 vs. Group 1	0.23	0.09	0.27	0.08	NS
Group 4 vs. Group 2	0.23	0.09	0.29	0.09	NS
Group 5 vs. Group 1	0.28	0.11	0.27	0.08	NS
Group 5 vs. Group 2	0.28	0.11	0.29	0.09	NS
Brain visfatin					
Group 1 vs. Group 2	0.67	0.15	0.61	0.13	NS
Group 3 vs. Group 1	0.56	0.19	0.67	0.15	NS
Group 3 vs. Group 2	0.56	0.19	0.61	0.13	NS
Group 4 vs. Group 1	0.45	0.11	0.67	0.15	<0.001
Group 4 vs. Group 2	0.45	0.11	0.61	0.13	<0.01
Group 5 vs. Group 1	0.56	0.18	0.67	0.15	NS
Group 5 vs. Group 2	0.56	0.18	0.61	0.13	NS

* NS-statistically not significant.

## Data Availability

Data is available on request from the authors.
